# Risk of neuropsychological impairment among therapeutic community residents: relationship with dropout and spontaneous recovery during treatment

**DOI:** 10.1186/s12888-026-07995-1

**Published:** 2026-03-21

**Authors:** Simon Deniel, Marion Delarue, Louise Quételard, Maïlys Miquel, Stéphane Lozé, Marie Dumont, Christophe Rimbaud, Susie Longbottom, Nicolas Kenens, Nicolas Bourguignon, Jonathan Rayneau, Juliette Dupuis, Marine Gaubert, Hélène Beaunieux, Ludivine Ritz

**Affiliations:** 1 Université de Caen Normandie, LPCN UR 7452, Caen, F-14000 France; 2 Communauté Thérapeutique de la Sauvegarde du Nord, Le Cateau- Cambrésis, France; 3 Communauté Thérapeutique du the Fleuve, CEID Addictions, Barsac, France; 4 Communauté Thérapeutique d’Aubervilliers, Aurore Association, Aubervilliers, France; 5 Federation Addiction, Paris, France; 6 UFR de Psychologie, Bâtiment L, Esplanade de la Paix, Caen Cedex 5, 14032 France

**Keywords:** Substance use disorders, Polysubstance use, Therapeutic communities, Risk of neuropsychological impairment, Dropout, Cognitive recovery, Ataxia, Cognitive screening

## Abstract

**Background:**

Therapeutic Communities (TCs) are long-term residential treatment settings for individuals with substance use disorders, who often present with neuropsychological impairments. These impairments may influence treatment retention, yet little is known about their evolution in TCs or their association with dropout. This study aims to: (1) identify early risk factors for dropout; (2) investigate the potential recovery from the risk of neuropsychological impairment during an 8-mont stay in a TC; and 3) identify variables at TC entry that may predict the risk of neuropsychological impairment during follow-up.

**Methods:**

Fifty-seven residents from 3 French TCs completed clinical, substance use, and medical assessments, along with a neuropsychological screening (BEARNI) at entry. Twenty-four completed the 8-month follow-up.

**Results:**

The dropout rate was 47%. The only significant predictor of dropout was the BEARNI total score, with residents at lower risk of neuropsychological impairment more likely to leave the TC prematurely. Among completers, BEARNI total scores improved at follow-up, primarily explained by improvements in balance rather than in cognitive areas. Lower education, unemployment, recent alcohol use, depressive symptoms, and a history of hepatic disease were consistently associated with a higher risk of neuropsychological impairment at both time points.

**Conclusions:**

Unlike other treatment settings, the TC care framework may be particularly appropriate and supportive for residents who are more cognitively vulnerable, as they may be better able to benefit from TCs. Motor recovery among those who remain in treatment is encouraging. However, demographic, addiction-related, and comorbidity factors are consistently associated with the risk of neuropsychological impairment, both at baseline and after 8 months, suggesting a stable and persistent influence over time.

**Clinical trial number:**

Not applicable.

**Supplementary Information:**

The online version contains supplementary material available at 10.1186/s12888-026-07995-1.

## Background

Therapeutic Communities (TCs) are residential addiction treatment facilities that welcome individuals with substance use disorders (i.e., residents) and complex comorbidities who are ready to engage in dynamic change [1]. In France, TCs are relatively few [2] and typically offer a highly structured 12- to 24-month residential program based on communal living, gradual responsibilisation, close professional supervision, and a focus on reintegration into society. This approach enables residents to benefit from the expertise of their more experienced peers, fostering interaction skills, self-help, and the experience of living without substances. TCs are of great interest both experimentally and clinically, as they offer the opportunity to follow-up patients with substance use disorders (SUD) and multiple comorbidities [3], and to study a population that is typically excluded from research. In addition, this therapeutic model allows for long-term follow-up and the examination of various factors associated with the treatment process and the risk of neuropsychological impairment. Indeed, residents in TCs are often polysubstance users with long and ineffective treatment histories. They commonly present with psychiatric comorbidities [4–6], all of which have been associated with poor cognitive functioning (including deficits in attention, working memory, executive functions, and social cognition) [7, 8], as well as a high risk of dropout [3, 9]. Some studies have taken advantage of this clinical variability through a naturalistic approach (without specific inclusion or exclusion criteria) to identify profiles at the highest risk of poor cognitive functioning [4, 10], dropout [11], or low recovery [12].

Studies conducted in TCs using a naturalistic approach have highlighted the high prevalence of cognitive impairments among residents, affecting almost half of them [13]. These impairments are mainly characterized by executive dysfunction, with memory difficulties appearing less prominent [10]. The risk of cognitive impairment is higher among residents with a history of traumatic brain injury [13] and constitutes a poor prognostic factor for both treatment retention and engagement in the program [14].

These findings are consistent with studies conducted in other settings that excluded comorbid conditions in order to specifically isolate the effects of substance use on neuropsychological functioning. They consistently show that substance use leads to cognitive impairments, the nature and severity of which vary depending on the substance involved. These studies confirm the high prevalence of cognitive disorders in SUDs (32–43%; [14, 15]), particularly executive dysfunction [4, 13, 16]. Other studies have also reported deficits in working memory and episodic memory, albeit with lower prevalence [16, 17]. In polysubstance use disorders, studies have demonstrated impairments in executive functions [18], working memory [19], and episodic memory [20]. In TC residents, a single study assessed various cognitive domains using a retrospective computer-based approach, revealing alterations in attention, processing speed, and verbal memory [10].

The presence of cognitive impairments may be associated with unfavorable treatment outcomes, such as poor adherence to treatment, early dropout (approximately 30% in SUD care), and high relapse rates (up to 80%) [21]. Thus, treatment adherence must be considered, as preventing dropouts has been linked to improved health and wellbeing [22]. Studies focused on identifying risk factors for dropout in SUD treatment have found that cognitive impairments affecting inhibition, attention, flexibility, and abstract reasoning are among the most frequently reported factors [23, 24]. The influence of alcohol use severity, substance use, and psychiatric comorbidities on the risk of dropout has also been documented, but remains more controversial [6, 25].

Studies have shown that the dropout rate is significantly higher in TCs and long-term inpatient programs than in short-term programs [25, 26], ranging from 6% to 74% across studies [11, 26, 27]. In the context of TCs, residents’ demographic characteristics (age, sex, and education [6, 11]), higher levels of stress at entry [5], a history of crack use [6], previous treatment history [11], and cognitive deficits [28] have been associated with higher dropout rates. The influence of primary substance use and psychiatric disorders on dropout is more inconsistent [27].

Despite these previous findings, studies conducted in the context of TCs have examined demographic, substance use, medical history, and neuropsychological variables separately rather than in combination. The combination of these variables may represent high-risk factors for dropout and should be carefully considered, particularly in TCs, where they are prevalent. Importantly, although these factors contribute to poorer clinical outcomes, cognitive impairments in patients with SUD are not necessarily permanent. In fact, recovery can manifest as improved performance relative to baseline or complete normalization [29]. Studies conducted in SUD and polysubstance use disorders have reported improvements in executive functions, episodic memory, working memory, and visuospatial abilities [20, 30]. However, the degree of reversibility varies considerably depending on the cognitive function, ranging from six weeks to several years [17, 31–33]. One possible explanation is the methodological variation between studies, given that cognitive profiles are initially highly heterogeneous in SUD [34], and that cognitive functions do not recover equally, with each function following a different course. In addition, studies differ in whether they control for comorbidities, which may contribute to poorer cognitive recovery in residents with additional psychiatric or medical conditions. To date, only one study has examined changes in executive functioning in TC residents and found no significant changes after 4 weeks of treatment [4]. The small number of neuropsychological studies conducted in TC facilities is largely due to the absence of neuropsychologists in these settings, particularly in France. Introducing a neuropsychological approach through staff training in cognitive screening is a first step with both clinical and research value, enhancing our understanding of the risk of neuropsychological deficits, their clinical impact, and recovery in complex forms of SUD. Furthermore, using a clinical tool to collect research data ensures better generalizability among practitioners.

Few studies have simultaneously examined the role of demographic characteristics, substance use, comorbidities (liver, neurological, and psychiatric conditions), psychological state, and the risk of neuropsychological impairment at entry on dropout in TCs. Identifying profiles at risk of early dropout is essential for improving treatment adherence, tailoring the care pathway, and providing the most suitable accommodations. In addition, to the best of our knowledge, no follow-up studies are currently available that investigate changes in the risk of neuropsychological impairments in TCs.

For the first time in TCs, this study aimed to achieve three primary objectives using a naturalistic approach: (1) identify variables that may serve as risk factors for early dropout among TC residents, (2) estimate the potential recovery of the risk of neuropsychological impairments related to SUD withdrawal during the 8-month follow-up period within TCs, and (3) identity variables at TC entry that may predict changes in the risk of neuropsychological impairments during follow-up. Based on existing literature, it was expected that (1) early dropout would be associated with addiction history, cognitive deficits, and psychiatric and medical history; (2) the TC context would reduce the risk of neuropsychological impairment during follow-up, due to abstinence-related improvements in cognitive and motor functions; and (3) addiction and psychiatric history may have a detrimental effect on recovery from the risk of neuropsychological impairment during follow-up.

## Methods

This study was conducted as part of a longitudinal research protocol (known as Neuropsychology of Addictions in Therapeutic Communities, NeuroAddiCT), which aims to evaluate the effectiveness of a neuropsychological intervention in TCs on relapse rates and social-professional reintegration [35]. This article focuses on the control group of the project, which aims to assess spontaneous cognitive changes during long-term TC stays, without integrating neuropsychological care into the treatment pathway.

### Participants

**Fig. 1:**
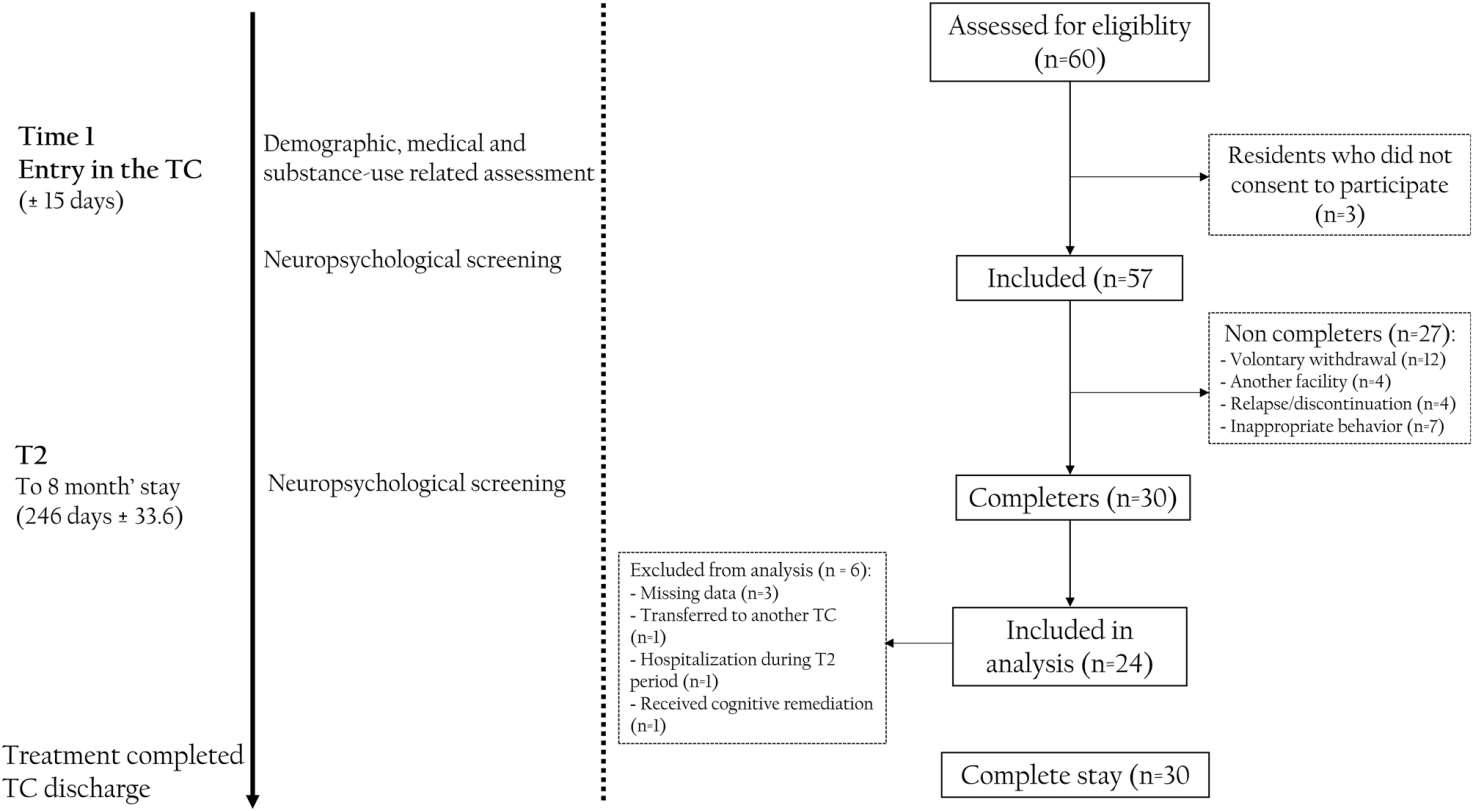
CONSORT-style diagram of the experimental design. Sixty participants were eligible for inclusion and three declined to participate. Fifty-seven participants were included at entry to the Therapeutic Community (± 15 days). During the stay, 27 of them left the TC before the end of treatment and did not undergo neuropsychological screening at Time 2. Thirty participants remained in the TC, and 24 of them underwent neuropsychological assessment at Time 2

Fifty-seven volunteer participants were included consecutively within 15 days of their entry into 3 French TCs participating in the research program (TC1: Communauté Thérapeutique d’Aubervilliers; TC2: Communauté Thérapeutique du Cateau Cambrésis; TC3: Communauté Thérapeutique du Fleuve à Barsac). Participants entered the TCs voluntarily, often after repeated treatment failures and following guidance from medical or social care providers. No a priori power calculation was performed, as the sample size was determined by the natural flow of admissions over the inclusion period. Following the approach of [10, 13], a naturalistic methodology was used to include all volunteer participants, closely reflecting the clinical reality of TCs. This methodology was used to capture the demographic, substance use, and comorbid profiles of participants. Each resident presented with SUD, was a native French speaker, had at least seven years of formal education, and possessed basic literacy and numeracy skills. They underwent two assessments using the same procedure at different time points: the first assessment was conducted within 15 days of entry into the TCs (Time 1; *n* = 57), and the second assessment occurred at an 8-month follow-up during their stay (Time 2; *n* = 24) (see Fig. 1 for the CONSORT-style flow diagram of participant inclusion and follow-up).

For clarity, the term “participants” is used to refer exclusively to individuals enrolled in the present study, whereas the term “residents” refers to the broader TC population.

### Ethics

The study was approved by the Research Ethics Committee (CLERS) of the University of Caen (approval number 3627). All participants were informed about the study (its purpose and data collection) prior to their inclusion and provided their written informed consents. The Ethical Principles of Psychologists and Code of Conduct of the American Psychological Association [36] regarding the ethical treatment of human participants were respected for all participants.

### Measures

All relevant measures concerning the TC participants are presented in Table 1.

**Table 1:** Baseline characteristics of TC participants assessed at Time 1, for the whole sample and for the subgroup who completed Time 2

Variables	Participants at Time 1 (*n* = 57)	Participant who completed Time 2 (*n* = 24/57)
***Demographic and clinical characteristics***		
Sex** (M/F) %	45/12 79% / 21%	20/4 83% / 17%
Age (in years) * *Range*	41.00 ± 10.20 *20–57*	41.8 ± 12.20 *20–57*
Years of schooling* *Range*	11.40 ± 2.19 *7–17*	10.90 ± 1.96 *7–17*
Alcohol abstinence (days) * *Range*	398 ± 1882 7-14274	724 ± 2891 9–14,274
Living environment** (Stable/Precarious) %	46/11 81% / 19%	21/3 87% / 13%
Employment before treatment ** (Employed/Unemployed) %	12/45 21% / 79%	6/18 25% / 75%
Substance Use**		
Tobacco use in the last 12 months (Users/Non users) %	55/2 96% / 4%	24/0 100%
Alcohol use in the last 12 months (Users/Non users) %	50/7 88% / 12%	21/3 87% / 13%
Cannabis use in the last 12 months (Users/Non users) %	34/23 60% / 40%	13/11 54% / 46%
Heroin use in the last 12 months (Users/Non users) %	7/50 12% / 88%	3/21 13% / 87%
Benzodiazepines use in the last 12 months (Users/Non Users) %	30/27 53% / 47%	15/9 62% / 38%
Polysubstance use (Yes/No) %	48/9 84% / 16%	21/3 87% / 13%
Medical history **		
Liver history (Yes/No) %	25/32 44% / 56%	10/14 42% / 58%
Neurological history (Yes/No) %	41/16 72%/ 28%	16/8 67% / 33%
History of traumatic brain injury (Yes/No) %	23/57 40% / 60%	8/24 33% / 67%
Psychiatric history (Yes/No) %	50/7 88% / 12%	21/3 87% / 13%
Serious mental illness^1^ (Yes/No) %	13/44 23% / 77%	3/21 12.5% / 87.5%
Depression/anxiety (Yes/No) %	30/27 53% / 47%	17/7 71% / 29%
Psychopathological state *		
Anxiety score (HADS-A) *Range*	10.50 ± 4.65 *2–21*	9.63 ± 4.02 *2–17*
Depression score (HADS-D) *Range*	6.21 ± 3.38 *1–15*	5.71 ± 3.01 *1–10*
Neuropsychological screening*		
BEARNI total score *Range*	14.50 ± 5.01 *5.5–27.5*	13.60 ± 4.36 *7.5–20.5*

Note. Data are presented as * mean ± standard deviation or ** *n %*

BEARNI: Brief Evaluation of Alcohol-Related Neuropsychological Impairments; HADS: Hospital Anxiety and Depression Scale

^1^ serious mental illness: schizophrenia or psychosis

#### Demographic, medical, and substance use-related variables

At time 1, ad hoc interviews conducted by TC clinicians collected data on age, sex assigned at birth (male or female), years of education, living environment (stable or precarious), and employment status prior to TC entry (employed or unemployed). The interviews also documented substance use, focusing on alcohol, tobacco, cannabis, heroin, and benzodiazepines. All substance use measures, including Approximate Lifetime Consumption, were quantified using a modified version of the semi-structured interview developed by Pfefferbaum et al. [37]. For each life period, participants reported the average quantity consumed per day and the number of days of use per week. These data were then used to determine whether participants had consumed substances in the 12 months preceding TC entry. Polysubstance use was also documented (defined as the consumption of at least two substances simultaneously during the past 12 months, excluding tobacco). The number of days of alcohol abstinence prior to TC entry was also recorded. Medical history variables were documented by physicians (yes/no) and included liver, neurological (including traumatic brain injury, stroke or other neurological diseases), and psychiatric histories (including depression, anxiety, schizophrenia, and borderline personality disorder). The assessment of participants’ current psychopathological state (e.g., anxiety and depression) was conducted using the Hospital Anxiety Depression Scale (HADS) [38]. The HADS was chosen as it is a brief, well-validated tool, widely used in addiction services, allowing for the rapid and reliable assessment and follow-up of anxiety and depressive symptoms [39].

#### Neuropsychological screening – BEARNI

The risk of neuropsychological impairments was assessed at both time points using the Brief Evaluation of Alcohol-Related Neuropsychological Impairments (BEARNI) [40]. The assessment takes approximately 20 min to administer. A parallel version of the test was used at Time 2 to facilitate follow-up assessments and the evaluation of potential cognitive recovery. This screening tool was designed to identify the risk of cognitive and motor deficits (i.e., executive function, episodic memory, working memory, visuospatial abilities, and ataxia) in patients with alcohol use disorders (AUD). It consists of five subtests: a delayed verbal episodic memory subtest (maximum score: 6 points), an alphabetical span subtest assessing verbal working memory (maximum score: 5 points), an alternating verbal fluency subtest assessing cognitive flexibility (maximum score: 6 points), a complex figures subtest assessing visuospatial abilities (maximum score: 5 points), and an ataxia subtest assessing balance (maximum score: 8 points). Domain subscores have been shown to predict performance on an extended neuropsychological battery [40], while also providing insight into participants’ cognitive and motor profiles and the evolution of spontaneous recovery.

The BEARNI yields six scores: five subscores and one total score (maximum score: 30 points). Lower scores indicate poorer cognitive and motor performance. For participants with 12 years of education or fewer, a total score of 16 or below indicates a risk of moderate to severe neuropsychological impairment [40]. The validated cut-off scores adjusted for years of education were used to estimate the prevalence of neuropsychological risk in the sample.

Given the high prevalence of alcohol use in TCs [5, 25] and the lack of validated French-language screening tools for this population, the BEARNI was considered a relevant instrument for assessing the risk of neuropsychological impairment. In addition, it has demonstrated sensitivity to spontaneous cognitive recovery, which is particularly valuable in longitudinal studies [29].

### Data analysis

Descriptive statistics were used to characterize the demographic characteristics, substance use, medical history, and psychopathological state of TC participants.

#### Identification of baseline risk factors associated with dropout

To identify risk factors for dropout, a survival analysis was conducted on all participants at Time 1 (*n* = 57). The number of days since the residents arrived at the TCs was recorded and coded as the “Time elapsed” variable. Participants were followed until they left the TC, either due to premature dropout or completion of their treatment.

Multivariable survival analysis using Cox proportional hazards modelling was conducted to identify the potential effects of demographic, medical, and substance use-related variables, as well as the BEARNI total score at Time 1 (described in Table 1), on length of stay, adjusting for TCs. The observed events were participants who dropped out. Due to the large number of variables, only those with an univariable p value ≤ 0.05 were included in the final model. The cut-off used in the Cox model was derived using maximally selected rank statistics and was not interpreted as a diagnostic threshold.

#### Changes in the neuropsychological screening measure at follow-up

To assess changes in the risk of neuropsychological impairments during participants’ stay in TCs, mixed-effects models were conducted on BEARNI scores (the BEARNI total score and the score of each subtest), with Time (T1 vs. T2) as a fixed effect, while accounting for random effects (individual differences among participants) and length of stay. Due to the high variability in alcohol abstinence among participants (Table 1), this variable was included as a covariate. Analyses were conducted on participants who remained in the TC after 8 months (*n* = 24).

#### Identification of baseline variables predicting changes in the neuropsychological screening measure over follow-up

Variables measured at T1 (demographic, medical, and substance use-related variables described in Table 1) were added to the mixed-effects model to investigate their influence on the BEARNI total score at Time 1 and Time 2. A stepwise selection procedure based on the Akaike Information Criterion (AIC) was applied. Covariates that did not contribute significantly to model fit were removed sequentially to obtain a parsimonious final model. Potential interactions between Time and the selected covariates were tested to determine whether the effects of these variables differed between T1 and T2. A full model including interactions was initially fitted, and non-significant interaction terms were removed using the AIC-based selection procedure. This approach aimed to determine whether the combination of variables at TC entry had a significant effect on changes in the risk of neuropsychological impairments between T1 and T2. As some categorical predictors had unbalanced distributions (e.g., low counts at certain levels; see Table 1), a Bayesian approach was used to improve the stability of the estimates. Results are reported with 95% credible intervals and directional probabilities (e.g. Pr(β > 0)).

The analysis was conducted using Jamovi and R, with statistical significance determined at an alpha level of 0.05.

## Results

Among all TC participants included (*n* = 57), there was a higher proportion of males, middle-aged, and those with years of schooling close to a high school diploma (Table 1). Most participants were unemployed before entry, but their living environment was stable. TC participants had a high prevalence of psychiatric, neurological, and liver issues in their medical history. Among the substances frequently used upon entry to the TCs, tobacco was the most common, followed by alcohol, cannabis, benzodiazepine misuse, and heroin. A significant majority of the sample engaged in polysubstance use (i.e., the simultaneous use of 2 or more substances, excluding tobacco, over the past 12 months). The duration of alcohol abstinence showed high variability among participants, highlighting considerable heterogeneity in drinking histories. Regarding their psychopathological state upon entry, 70% of participants scored above 7 on the HAD scale (i.e., indicating anxious symptomatology), and 29% exceeded the depression symptomatology cut-off score of 7.

At TC entry, 67% of participants had a BEARNI total score indicating moderate to severe impairment [40]. Regarding the BEARNI subtests, 53% were at risk for episodic memory impairments, 49% for ataxia, 3.5% for flexibility deficits, 61% for working memory impairments, and 23% for visuospatial deficits. These proportions were calculated using the validated BEARNI cut-offs.

### Risk factors for dropout in TCs

Comparisons between completers and dropouts revealed no significant differences in demographic characteristics, substance use, medical history, or baseline anxiety and depression scores (all p values > 0.05; Supplementary Table S1).

Between Time 1 and Time 2, 47% of the sample dropped out of the TCs before completing their treatment (*n* = 27), with a median duration of stay of approximately 130 days (± 115 IQ) for participants who dropped out before Time 2.

Results of the univariable cox regression analysis showed that only the BEARNI total score reached significance (Table 2). Although the effect of TC1 compared with TC3 was significant, the 95% confidence interval of the hazard ratio (HR) was wide and should be interpreted with caution. The comparison between TC1 and TC2 was not statistically significant; therefore, the TC variable was not included in the final model.

**Table 2:** Univariable analysis of length of stay among TC participants

Variables	HR	95% CI	*p* value
TC:			
TC1			
TC2	0.51	0.11–2.48	0.41
TC3	2.52	1.05–6.05	0.04*
Sex:			
Male			
Female	1.31	-0.53-3.25	0.56
Age	0.99	0.96–1.03	0.76
Years of schooling	1.12	0.94–1.33	0.21
Alcohol abstinence	1.00	0.99-1.00	0.62
Living environment:			
Stable			
Precarious	1.36	0.55–3.38	0.51
Employment before treatment:			
Employed			
Unemployed	0.89	0.36–2.21	0.80
Tobacco use in the last 12 months:			
Users			
Non users	0.45	0.11–1.94	0.29
Alcohol use in the last 12 months			
Users			
Non Users	1.37	0.41–4.55	0.61
Cannabis use in the last 12 months			
Users			
Non Users	1.17	0.54–2.57	0.69
Heroin use in the last 12 months			
Users			
Non Users	1.26	0.44–3.65	0.67
Benzodiazepines use in the last 12			
Users			
Non Users	0.57	0.27–1.23	0.15
Polysubstance use			
No			
Yes	1.00	0.35–2.89	0.98
Liver history			
No			
Yes	1.21	0.57–2.57	0.62
Neurological history			
No			
Yes	1.25	0.53–2.97	0.60
Psychiatric history			
No			
Yes	1.28	0.39–4.27	0.68
Anxiety score (HADS-A)	1.00	0.92–1.08	0.98
Depression score (HADS-D)	0.98	0.88–1.10	0.68
BEARNI total score	1.10	1.02–1.19	0.01*

TC1: Communauté Thérapeutique d’Aubervilliers; TC2: Communauté Thérapeutique du Cateau Cambrésis; TC3: Communauté Thérapeutique du Fleuve à Barsac

BEARNI: Brief Evaluation of Alcohol-Related Neuropsychological Impairments; HADS: Hospital Anxiety and Depression Scale

The analysis was conducted using survival analysis with Cox proportional hazards modelling

Results are presented as Hazard Functions (95% Confidence Intervals)

* significant at *p* ≤ 0.05)

**Fig. 2:**
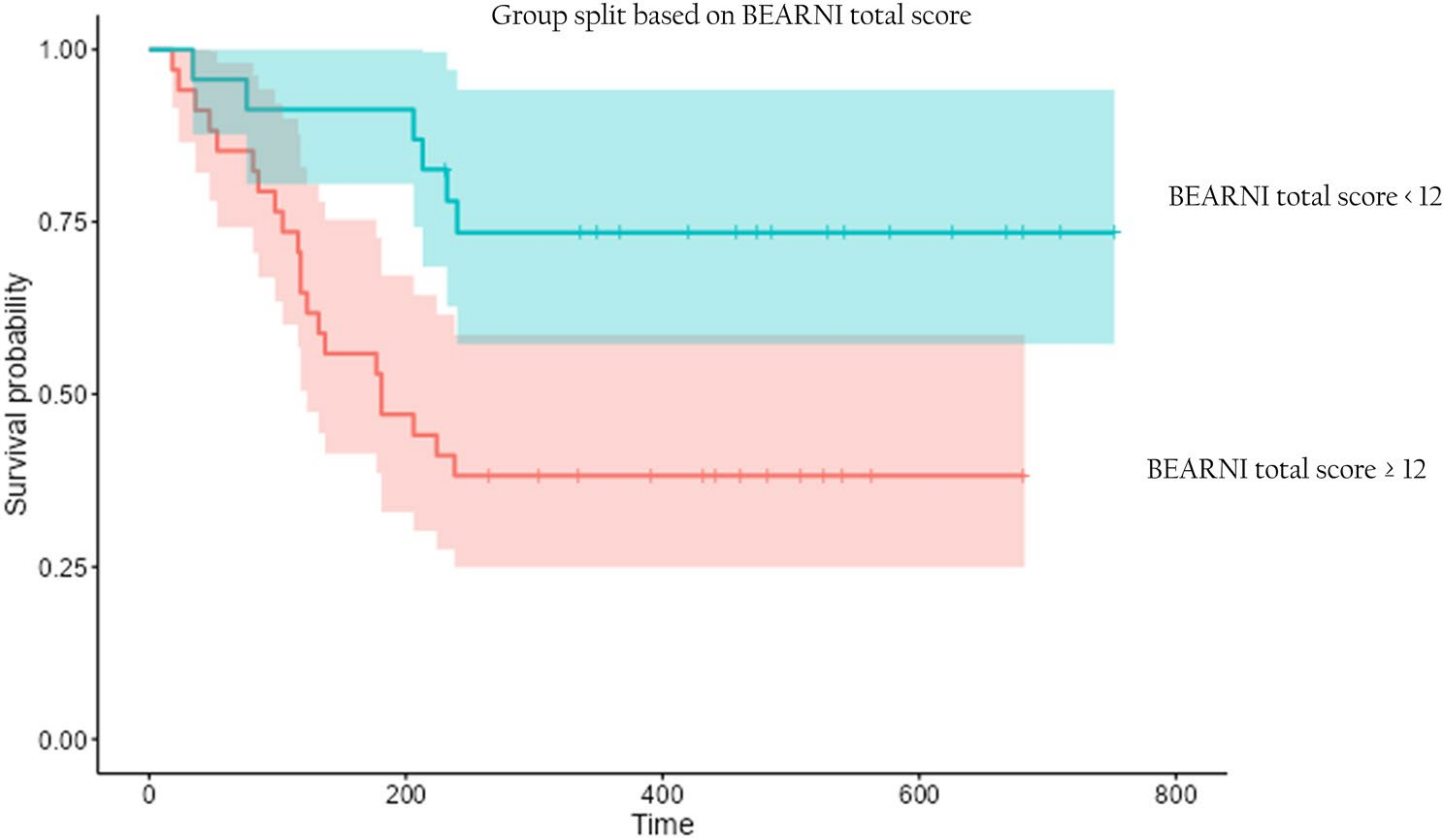
Survival plot of length of stay for dropout participants according to the BEARNI total score at Time 1. *n* = 57. Time in days. The red curve represents a high BEARNI total score at Time 1 (with 95% CI in lighter red), and the blue curve represents a low BEARNI total score at Time 1 (with 95%CI in lighter blue). The cut-off for the BEARNI total score at Time 1 was defined as 12 points

The BEARNI total score at Time 1 was the only significant predictor of dropout, with a small effect size (R² = 9.8%). A higher BEARNI total score at Time 1 was associated with a shorter length of stay and an increased risk of dropout (Fig. 2). The cut-off point of 12 was empirically determined by the survival analysis as the threshold that maximized the separation between survival curves, allowing discrimination between groups with different lengths of stay. Because the normative BEARNI cut-offs are adjusted for educational level [40], education was included as a covariate in the Cox analyses; however, it did not reach statistical significance.

A model including the BEARNI subscores showed that no individual domain, including ataxia, independently predicted dropout (all p values > 0.05).

### Changes in a neuropsychological screening measure

For the participants who remained in the TCs (*n* = 30), data were missing for three participants: one was transferred to another TC, one was hospitalized during the planned T2 period, and one received cognitive remediation between Time 1 and Time 2 (Fig. 1).

**Fig. 3:**
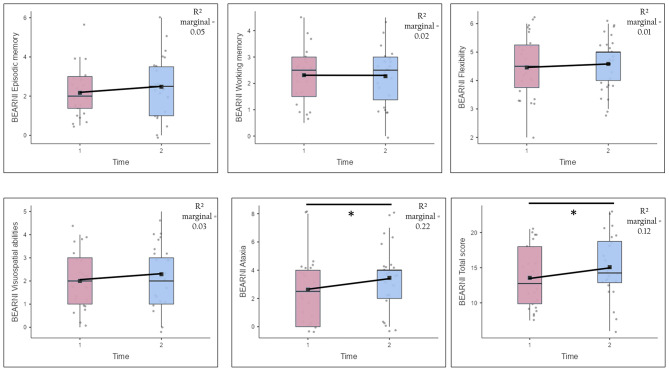
Distribution plots of BEARNI performance at Time 1 and Time 2. *n* = 24. Data are presented as mean ± standard deviation. Time 1 is represented in red, and Time 2 in blue. * significant difference between Time 1 and Time 2 at *p* ≤ 0.05. Mixed-effects models were conducted on BEARNI scores with Time (T1 vs. T2) as a fixed effect, accounting for random effects and length of stay, with alcohol abstinence as a covariate. R2 marginal represents the effect size of the fixed effect (= 0.01: small; ≤0.09: medium; ≤0.25: large)

The analysis of changes in the neuropsychological screening measure was performed only for participants who completed both Time 1 and Time 2 assessments (*n* = 24), and the results are presented in Table 3 and Fig. 3. The average length of stay was 491 ± 135 days [231–752]. These results indicate an increase in the average total BEARNI score at Time 2 compared to Time 1, with a medium effect size. Regarding the subtests, the only significant difference between Time 1 and Time 2 scores was observed in the ataxia subscore, which showed a medium effect size. None of the other subtests (i.e., episodic memory, visuospatial abilities, working memory, and flexibility) showed a significant change in performance at follow-up. The alcohol abstinence covariate was not significant (all p values > 0.05). Based on the BEARNI clinical cutoff (score ≤ 3), 58% of participants showed ataxia at entry compared with 42% after 8 months, indicating that a subset of individuals moved above the threshold for motor impairment.

**Table 3:** BEARNI performance scores for participants who completed both Time 1 and Time 2 assessments (*n* = 24)

BEARNI scores	Time 1	Time 2	Statistics^1^	Effect size (*R*^2^ marginal)
Total score *(max. score = 30)* *Range*	13.50 ± 0.66 *7.5–20.5*	15.10 ± 0.66 *6–23*	F_(1,23_) = 5.52; *p* = 0.03*	0.12
Episodic memory *(max. score = 6)* *Range*	2.19 ± 0.29 *0.5–5.5*	2.56 ± 0.29 *0–6*	F_(1,23_) = 1.37; *p* = 0.25	0.05
Working memory *(max. score = 5)* *Range*	2.28 ± 0.21 *0.5–4.5*	2.24 ± 0.21 *0-4.5*	F_(1,23_) = 0.05; *p* = 0.83	0.02
Flexibility *(max. score = 6)* *Range*	4.47 ± 0.23 *2–6*	4.60 ± 0.23 *3–6*	F_(1,23_) = 0.21; *p* = 0.65	0.01
Visuospatial abilities *(max. score = 5)* *Range*	1.99 ± 0.26 *0–4*	2.27 ± 0.26 *0–5*	F_(1,23_) = 1.27; *p* = 0.27	0.03
Ataxia *(max. score = 8)* *Range*	2.58 ± 0.44 *0–8*	3.44 ± 0.44 *0–8*	F_(1,23_) = 13.07; *p* < 0.001*	0.22

Data are presented as marginal mean ± standard error of the mean

^1^ Mixed-effects model; R2 marginal represents the effect size of the fixed effect (= 0.01: small effect size; ≤0.09: medium effect size; ≤0.25: large effect size)

* indicates a significant result at *p* ≤ 0.05

BEARNI: Brief Evaluation of Alcohol-Related Neuropsychological Impairments

Including the TC variable as covariate did not change the pattern of results and did not reach significance.

### Factors explaining changes in a neuropsychological screening measure

The final linear mixed-effects model, selected based on the AIC in a preliminary frequentist step, identified several covariates significantly associated with the BEARNI total score. The model included Time (T1 and T2), length of stay, the variables described in Table 1, and the TC, as fixed effects, with a random intercept for participants. To improve estimation stability given the small sample size and unbalanced distributions for some predictors, a Bayesian model was then used to estimate the effects of the selected variables. Results are presented as posterior means, 95% credible intervals, and directional probabilities. More years of schooling (β = 0.99, 95% CI [0.27; 1.72]; Pr(β > 0) = 0.994) were associated with higher BEARNI total scores, whereas a history of hepatic disorders (β = -3.01, 95% CI [-5.82; -0.19]; Pr(β > 0) = 0.980), alcohol use in the last twelve months (β = -6.49, 95% CI [-10.41; -2.43]; Pr(β > 0) = 0.999), higher HAD-D score (β = -0.52, 95% CI [-0.96; -0.06]; Pr(β > 0) = 0.986), and being unemployed prior to TC entry (β = − 4.89, 95% CI [-9.42; -0.45]; Pr(β > 0) = 0.983) were associated with lower BEARNI total scores. The final model provided a better fit than the initial full model, as indicated by a reduction in the AIC from 261.9 to 252.8. No significant interactions were retained, suggesting that the effects of these covariates on BEARNI total scores remained stable over time.

Post hoc power analyses were conducted using R software to assess the statistical sensitivity of the main models. The results indicated limited power for both the Cox regression (57 participants) and the mixed-effects models comparing T1 and T2 (24 participants with repeated measures), particularly for detecting small effects.

## Discussion

The results indicate that (1) dropout is frequent and predicted by a lower baseline risk of neuropsychological impairment, (2) participants who complete the treatment show a reduction in this risk, primarily in the motor domain, and (3) several clinical and psychosocial factors - including education, employment, psychiatric and hepatic history, and recent alcohol use - are consistently associated with the risk of neuropsychological impairment at both baseline and 8-month follow-ups. Although the post hoc power analyses indicate limited sensitivity to detect small effects, the study retains adequate power to identify effects of medium magnitude, such as improvements in ataxia. Nonetheless, given the modest sample size and the inherent attrition in long-term TC settings, the findings should be considered exploratory, and conclusions drawn with appropriate caution.

### Risk of neuropsychological impairment in TC participants

In this study, 67% of the participants were at risk of neuropsychological impairment, as estimated by BEARNI. This prevalence is consistent with previous studies conducted in TC using a naturalistic approach with a self-administered computer-based test battery [41], as well as with studies based on more selective samples of individuals with SUD [42–45]. Our results further confirm that cognitive impairments can be efficiently detected in TC residents using a brief screening tool, as demonstrated by Marceau et al. [13].

### Factors associated with dropout

In this study, nearly 50% of the participants dropped out before 8 months. This result is relatively consistent with studies reporting dropout rates between 45 and nearly 80% in TCs [27, 46]. Despite the high attrition rate in this study, no significant differences were observed between completers and non-completers regarding baseline demographic, substance use, or medical characteristics, suggesting that these factors alone are unlikely to account for the substantial dropout. This dropout rate has previously been related to various factors, such as cognitive impairments, which could significantly contribute to early departure, as seen in other addiction care settings [15, 47] and in TCs [14, 48]. However, contrary to our initial hypothesis, a higher score on the neuropsychological screening at entry appears to be associated with early dropout from the TC program. Our expectations were based on previous observations in SUD, which reported that impaired cognition could lead to low metacognitive abilities, resulting in reluctance to continue treatment and underestimation of the need for behavioral change [49]. These observations, however, are not corroborated in this study. One possible explanation for the discrepancy in results could relate to differences in the stage of cognitive recovery across studies. Although abstinence duration was included as a covariate in our analyses, variations in recovery may persist between samples, depending on the duration and pattern of substance use. Cognitive improvements in SUD patients are commonly observed during the first weeks following acute withdrawal [29, 32] and over the subsequent months [33], but tend to plateau after one year [50]. At the time of assessment, a significant proportion of our participants had exceeded the typical recovery window for substance-related cognitive impairments. Any remaining deficits were therefore likely residual and potentially long-lasting, possibly associated with comorbid neurological or psychiatric conditions, as previously observed by Marceau et al. [13]. An alternative explanation may be that individuals with long-standing and severe SUD, who consequently present with more pronounced and possibly enduring cognitive impairments, are also those most in need of care and possibly more motivated to remain in treatment. Their awareness of these difficulties may be heightened due to their long and complex treatment history. This awareness could act as a motivating factor for staying in the structured environment of a TC, which may explain the observed association between risk of neuropsychological impairments and dropout rate in our study. Other factors, which are not entirely unrelated to cognition, may act as barriers to treatment for drug users. These include treatment confidence and expectations, coping strategies, early alliance with the therapist, and previous treatment history [6, 46, 51]. These studies highlight the importance of the therapeutic alliance in drug treatment, as alliance ratings were found to be among the strongest predictors of dropout.

In our study, the association between a higher risk of neuropsychological impairment and a greater likelihood of remaining in the program could be attributed to the TC itself. TCs provide a highly structured environment, with strong environmental stability and staff available 24 h a day [52]. It has been shown that length of stay in TCs is related to increases in psychological, social, and emotional well-being [12]. However, such a highly structured daily life for 13 months or more (i.e., as proposed in French TCs) may not be suitable for every type of resident. Clinicians are regularly advised to support individuals with cognitive impairments by setting adjusted goals and guiding them step by step through a structured treatment, including rituals and a regular schedule, especially in cases of executive function and memory impairments [53]. This is what TCs can provide through their therapeutic framework, creating a reassuring sense of familiarity. This structured and secure environment may be particularly beneficial for cognitively vulnerable residents who require it for their recovery and the stabilization of comorbidities. However, it can be too demanding and less relevant for some residents who are cognitively intact, who may perceive the TC living framework as constraining, frustrating, or infantilizing, potentially leading them to feel as though they are putting their lives on hold [54]. Additionally, conflicts may arise in a system that relies on social sharing and self-identification among individuals who are less cognitively vulnerable and who do not identify with their cognitively impaired counterparts. Therefore, it is plausible that people with cognitive vulnerabilities may perceive or experience greater benefits from remaining for an extended period on their path to recovery, compared with their cognitively intact peers. Furthermore, the therapeutic alliance may also play a central role in this dynamic [6, 51]. Residents with cognitive vulnerabilities often require more guidance and support, which can foster closer interactions with staff and peers. This intensified relational engagement may strengthen their sense of belonging within the TC and contribute to their decision to remain in treatment.

### Recovery

The idea that a significant proportion of our study’s participants are beyond the typical recovery window for substance-related cognitive impairments is supported by the eight-month follow-up data, which showed no improvement in cognitive domains but a notable improvement in ataxia symptoms. Indeed, our results indicate an average improvement at the 8-month follow-up compared with baseline among the participants who remained in the TCs. However, this change was mainly driven by significant recovery in ataxia, whereas episodic memory, working memory, visuospatial abilities, and cognitive flexibility showed no significant improvement. This finding is consistent with previous studies highlighting heterogeneous and often limited recovery trajectories in polysubstance users with psychiatric or medical comorbidities [17, 55], in contrast to the more favorable outcomes typically reported in alcohol-only populations [30, 56, 57]. Indeed, potential recovery in polysubstance users is not clear-cut, with reports ranging from very little improvement [58] to no recovery at all over periods of 5 weeks to 1 year [59], and cognitive fragilities persisting even after 1 year [55]. In our study, despite a significant improvement in the BEARNI total score over the 8-month follow-up, 66% of the participants still presented scores indicating a moderate to severe risk of neuropsychological impairment based on the cut-off value. The high prevalence of comorbidities among TC residents may have long-term effects on cognitive functioning, which may be slowly reversible or even permanent, and may interact with substance-related consequences or premorbid vulnerabilities, making causal attribution particularly challenging. Recovery from ataxia may represent the final stage of recovery associated with abstinence. Indeed, ataxia is a specific neuropsychological deficit related to alcohol consumption and one of the most severe observed in patients with AUD [74, 75]. In our sample, 88% of participants had AUD. The severity of this impairment explains why recovery takes longer than that from alcohol-related cognitive deficits, in accordance with studies conducted on AUD patients [60, 61]. It is noteworthy that despite a significant improvement in the BEARNI ataxia score, 42% of the participants (compared with 58% at Time 1) still exhibited ataxia after 8 months of follow-up. The increase in mean ataxia scores is consistent with a partial but clinically meaningful recovery in balance. From a clinical perspective, improved balance is particularly relevant in the context of TCs as residential care settings, since greater autonomy of movement facilitates participation in daily activities and enhances independence. In addition, the improvement of such a visible impairment may increase motivation and adherence to treatment, as residents are better able to perceive the concrete benefits of abstinence and rehabilitation.

### Time-invariant risk factors for neuropsychological impairment

Some studies have investigated variables that could hinder cognitive recovery. In our study, demographic factors (education, professional status), an addiction-related factor (recent alcohol use), and comorbidity variables (history of hepatic disease and depressive symptoms) were found to influence the risk of neuropsychological impairment both at baseline and after 8 months of stay in the TC. Their effects remained stable over time, suggesting that these factors are consistently associated with the risk of neuropsychological impairment and may contribute to its persistence throughout the course of treatment.

More precisely, a low level of education is a risk factor for neuropsychological impairment, consistent with its influence on BEARNI total cut-off scores [40]. Lower education levels are modestly predictive of reduced cognitive recovery in individuals with SUD [30, 62]. Similarly, being unemployed prior to TC entry represents an additional risk factor, possibly reflecting broader socioeconomic disparities that may contribute to cognitive vulnerability. Regarding addiction patterns, in our sample only recent alcohol consumption (within the past year) had a deleterious impact on the risk of neuropsychological deficits [63], whereas numerous studies have found that a long history of alcohol use [64], substance use [32, 59, 65], benzodiazepine use [66], and tobacco use [67] are associated with an increased risk of neuropsychological impairment and poorer cognitive recovery. This finding is, however, consistent with others studies showing a higher neuropsychological risk in AUD patients with recent alcohol consumption [63]. In our sample, this effect may be explained by the wide variability in abstinence duration, despite a relatively long average. Finally, regarding medical comorbidities, a history of hepatic disease and depressive symptoms assessed at TC entry are associated with an increased risk of neuropsychological impairment at baseline and after 8 months. The impact of depression on cognition remains inconsistent, with some studies reporting no deleterious effects [68, 69], while others have found a higher risk of cognitive impairment in SUD patients with severe depression compared to those with moderate or no depression [70, 71]. Although liver disease is frequently observed in SUD patients [72–74] and has been associated with moderate neuropsychological impairment in AUD patients [75], the present study is the first to demonstrate a deleterious association between a history of liver disease and the risk of neuropsychological impairment at baseline and follow-up in TC residents.

It is important to note that these factors have a lasting influence on BEARNI total scores, as their effects persist over time without variation. This indicates that their impact on cognition is stable across the follow-up period, rather than being limited to a specific assessment point, and suggests that these factors represent stable determinants of the cognitive profile. In contrast to demographic factors and alcohol use prior to TC entry, which are non-modifiable and cannot be altered by treatment interventions, depressive symptoms and hepatic diseases can be managed. Given the high prevalence of depressive symptoms and hepatic comorbidities among TC participants at entry, early medical and psychological interventions are strongly recommended to reduce the risk of neuropsychological impairment. Finally, the chronicity of SUD, long-standing medical comorbidities, and repeated treatment failures in some residents may indicate that cognitive recovery has reached a plateau. The sustained impact of certain risk factors over time may reflect persistent cognitive impairments, highlighting the need for extended follow-up to determine whether further recovery is possible and, if not, to adapt clinical care accordingly.

### Clinical implications

Given that our findings indicate an association between baseline risk of neuropsychological impairment and treatment retention, routine neuropsychological screening at admission to TCs, using instruments such as the BEARNI, appears particularly relevant. Such screening should be complemented by a more comprehensive baseline assessment of medical history, substance use disorders, and current psychiatric status. Early identification and management of psychiatric comorbidities, such as depression, are particularly important, as our findings suggest that these factors adversely affect the risk of neuropsychological impairment at TC entry and after 8 months of residence. Detecting and addressing these factors early, for instance through tailored psychosocial interventions, may therefore help mitigate their impact on cognitive outcomes. Regarding hepatic conditions, abstinence from substance use and appropriate medical care in cases of chronic hepatic disease are essential to limit their potential negative effects on spontaneous neuropsychological recovery. In addition, re-assessment of neuropsychological risk during follow-up, for example using the parallel version of the BEARNI, could help monitor changes and guide treatment planning. For residents whose risk of neuropsychological impairment does not improve, extending the duration of TC stay may be beneficial. Finally, treatment approaches should be adapted to the cognitive profile of each resident: less rigid and more flexible structures may be appropriate for those with no or only mild cognitive vulnerabilities, whereas residents with more severe impairments may require a more structured and supportive environment, with a stronger emphasis on routines and individualized assistance.

### Limitations

This study has some limitations that should be considered. The BEARNI is a screening tool designed to predict the risk of neuropsychological impairments and does not provide a comprehensive cognitive assessment. It does not evaluate decision making, social cognition, or metacognition, which are domains frequently affected in individuals with SUD and highly relevant for treatment engagement and daily functioning in TCs. Because these functions were not assessed, some deficits may not have been detected, and the results should therefore be interpreted in light of this limitation. BEARNI also does not evaluate patients’ awareness of their cognitive difficulties, which limits the interpretation of certain findings. As a screening tool, it cannot differentiate the contribution of SUD-related factors from that of neurological comorbidities to the risk of neuropsychological impairment. This tool is intended to guide the need for a comprehensive neuropsychological assessment, and the present findings could potentially benefit from a more detailed cognitive examination. It is important to note that the BEARNI cut-offs indicate risk of impairment rather than diagnostic classification, and were therefore not used for prognostic modelling. The sample size was limited, partly due to the high attrition rate that is common in longitudinal studies of SUD. As a result, the statistical power was low, particularly for detecting small effects, and the analyses should be considered exploratory. However, the study retained sufficient sensitivity to detect effects of medium magnitude. The results should be viewed as preliminary patterns that require confirmation in larger samples. Moreover, the relatively small number of participants who remained in the TC precluded analyses of subgroups (e.g., substance use patterns) that might explain dropout or recovery, as well as factors influencing the lack of improvement on specific BEARNI subtests. It is therefore possible that other factors, which are not significant in the present study, may interact with dropout rates and cognitive recovery, opening new avenues for research.

The 8-month follow-up may also be too short to capture the full course of cognitive recovery, which can extend over longer periods of abstinence. Nevertheless, the sample size and attrition rate observed in this study are comparable to those reported in previous TC studies, supporting the relevance of our findings while highlighting the need for replication in larger cohorts with extended follow-up.

Another limitation is the absence of formal performance or symptom validity measures. In French medico-social settings, including TCs, neuropsychological screening is routinely conducted by physicians of healthcare staff who do not implement Performance Validity Tests/Symptom Validity Tests in standard practice. Although behavioral data were cross-checked with clinical interviews and medical records, these procedures do not replace formal validity testing. The BEARNI results should therefore be interpreted as screening indicators of risk rather than definitive evidence of neuropsychological impairment.

## Conclusions

In conclusion, the findings suggest that dropout is a common occurrence among TC participants, with a lower risk of neuropsychological impairment identified as a primary factor. However, the relationship between cognition and dropout was unexpected: participants with a higher risk of neuropsychological impairment tended to remain in TCs. The controlled and structured framework of TCs appears to be highly effective in supporting residents with moderate to severe cognitive impairment. Among those who stay, moderate recovery at 8 months is observable but limited to ataxia, while other cognitive domains show no significant improvement. For residents with severe and chronic clinical profiles, cognitive recovery may be limited or may not occur, highlighting the importance of long-term follow-up and adaptive treatment approaches. The high risk of neuropsychological impairment at entry, together with a history of hepatic disease and the presence of comorbid depression, underscores the need for a multidisciplinary approach. This should include early cognitive screening, medical treatment, and psychological support, all aimed at reducing the risk of neuropsychological impairments and promoting recovery.

## Supplementary Information

Below is the link to the electronic supplementary material.

Supplementary Material 1

## Data Availability

Data are available at: https://osf.io/9qp6g/.

## Abbreviations

AUD: Alcohol Use Disorder
SUD: Substance Use Disorder
TC: Therapeutic Community
NEUROADDICT: Neuropsychology of Addictions in Therapeutic Communities
HADS: Hospital Anxiety and Depression Scale
BEARNI: Brief Evaluation of Alcohol-Related Neuropsychological Impairments
