# Supplementary material for: Risk of neuropsychological impairment among therapeutic community residents: relationship with dropout and spontaneous recovery during treatment
Source: BMC Psychiatry. 2026 Mar 21;26:352. doi: 10.1186/s12888-026-07995-1 (PMC13126905; doi:10.1186/s12888-026-07995-1)
Supplement: Supplementary file 1 — Supplementary Material 1 [file 12888_2026_7995_MOESM1_ESM.docx]

Table S1: Baseline comparisons between completers and non-completers TC residents

| **Variables** | **Completers**  **(*n* = 30)** | **Non-completers**  **(*n* = 27)** | **Statistics; p value** |
| --- | --- | --- | --- |
| ***Demographic and clinical characteristics*** | | |  |
| Sex** (M/F) | 24/6 | 21/6 | Chi² = 0.04; 0.84 |
| Age (in years) * | 41.40 ± 11.03 | 40.59 ± 9.38 | t_(55)_ = 0.30; 0.77 |
| Years of schooling* | 11.00 ± 1.84 | 11.74 ± 2.51 | t_(55)_ = 1.28; 0.20 |
| Alcohol abstinence (days) * | 623.73 ± 2585.84 | 146.81 ± 215.08 | t_(55)_ = 0.95; 0.34 |
| Living environment** (Stable/Precarious) | 25/5 | 21/6 | Chi² = 0.28; 0.60 |
| Employment before treatment ** (Employed/Unemployed) | 6/24 | 6/21 | Chi² = 0.04; 0.84 |
| ***Substance Use***** | | |  |
| Tobacco use in the last 12 months (Users/Non users) | 30/0 | 25/2 | Chi² = 2.30; 0.13 |
| Alcohol use in the last 12 months (Users/Non users) | 26/4 | 24/3 | Chi² = 0.06; 0.80 |
| Cannabis use in the last 12 months (Users/Non users) | 17/13 | 17/10 | Chi² = 0.23; 0.63 |
| Heroin use in the last 12 months (Users/Non users) | 3/27 | 4/23 | Chi² = 0.31; 0.58 |
| Benzodiazepines use in the last 12 months (Users/Non Users) | 18/12 | 15/12 | Chi² = 1.38; 0.24 |
| Polysubstance use (Yes/No) | 25/5 | 23/4 | Chi² = 0.04; 0.85 |
| ***Medical history ***** | | |  |
| Liver history (Yes/No) | 12/18 | 13/14 | Chi² = 0.38; 0.54 |
| Neurological history (Yes/No) | 21/9 | 20/7 | Chi² = 0.12; 0.73 |
| Psychiatric history (Yes/No) | 26/4 | 24/3 | Chi² = 0.06; 0.80 |
| ***Psychopathological state **** | | |  |
| Anxiety score (HADS-A) | 10.53 ± 4.75 | 10.44 ± 4.64 | t_(55)_ = 0.07; 0.94 |
| Depression score (HADS-D) | 6.30 ± 3.34 | 6.11 ± 3.49 | t_(55)_ = 0.21; 0.84 |

*Note.* Data are presented as * mean ± standard deviation or ** *n*.

HADS: Hospital Anxiety and Depression Scale.


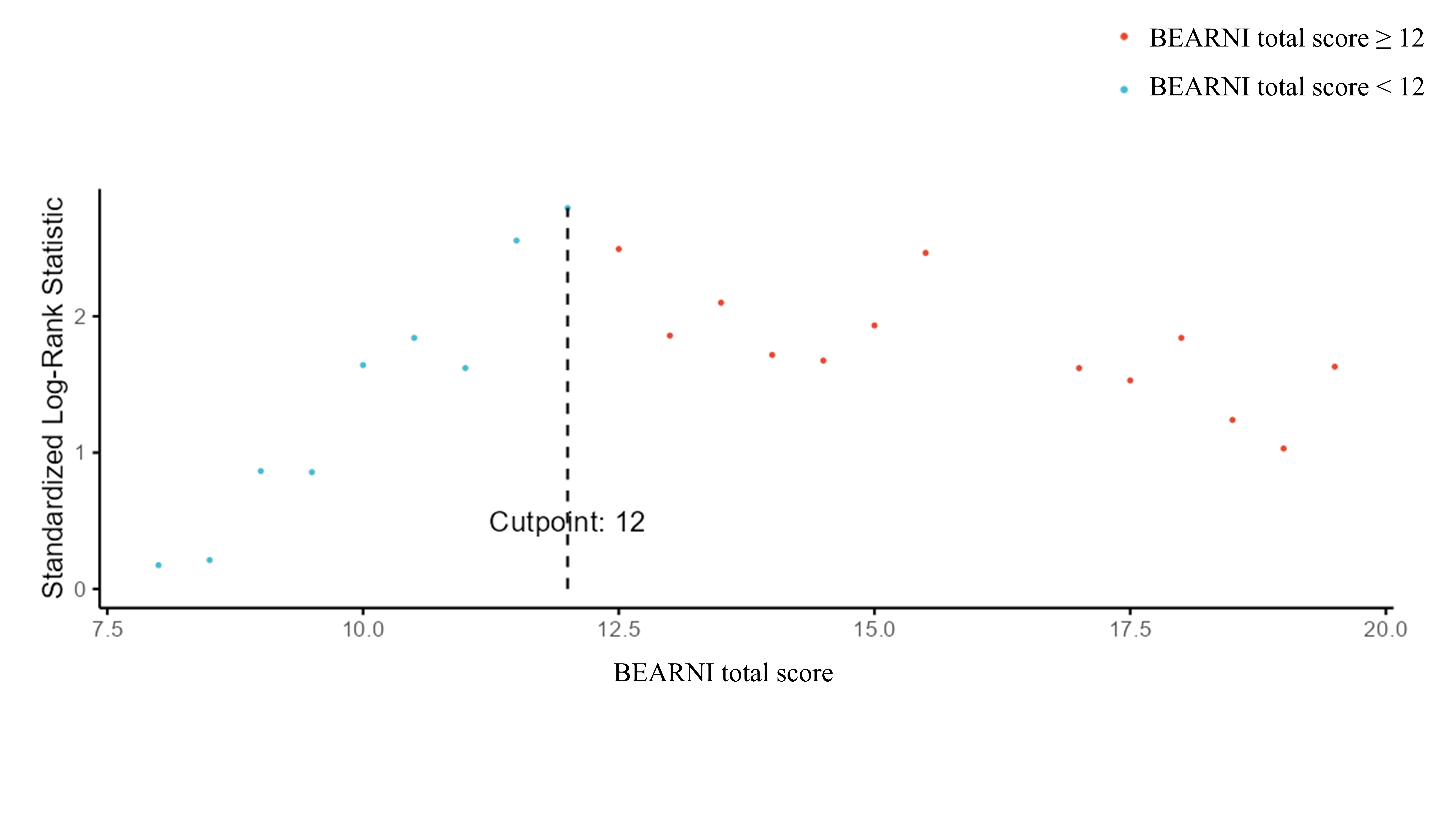


Figure S1: Determination of the optimal BEARNI cutoff using maximally selected rank statistics

The figure represents the maximally selected rank statistics used to determine the optimal cutoff for the BEARNI total score at Time 1. The vertical dashed line marks the value (12 points) at which the standardized log-rank statistics reaches its maximum, indicating the threshold that best separates participants according to their length of stay in treatment. This data-driven cutoff was subsequently used to define the two groups (BEARNI <12 vs BEARNI ≥12) in the Kaplan-Meir survival analysis.
